# Mutational pressure dictates synonymous codon usage in freshwater unicellular α - cyanobacterial descendant *Paulinella chromatophora* and β - cyanobacterium *Synechococcus elongatus* PCC6301

**DOI:** 10.1186/2193-1801-2-492

**Published:** 2013-09-30

**Authors:** Rahul Raveendran Nair, Manivasagam Bharatha Nandhini, Thilaga Sethuraman, Ganesh Doss

**Affiliations:** Department of Biotechnology, Vignan University, Vadlamudi, 522 213 Guntur, Andhra Pradesh India; Department of Plant Biotechnology, School of Biotechnology, Madurai Kamaraj University, Palkalai Nagar, 625 021 Madurai, Tamil Nadu India

**Keywords:** *Paulinella chromatophora*, *Synechococcus elongatus*, Synonymous codon usage, Mutational pressure, Chromatophore

## Abstract

**Background:**

Comparative study of synonymous codon usage variations and factors influencing its diversification in α - cyanobacterial descendant *Paulinella chromatophora* and β - cyanobacterium *Synechococcus elongatus* PCC6301 has not been reported so far. In the present study, we investigated various factors associated with synonymous codon usage in the genomes of *P*. *chromatophora* and *S*. *elongatus* PCC6301 and findings were discussed.

**Results:**

Mutational pressure was identified as the major force behind codon usage variation in both genomes. However, correspondence analysis revealed that intensity of mutational pressure was higher in *S*. *elongatus* than in *P*. *chromatophora*. Living habitats were also found to determine synonymous codon usage variations across the genomes of *P*. *chromatophora* and *S*. *elongatus*.

**Conclusions:**

Whole genome sequencing of α-cyanobacteria in the cyanobium clade would certainly facilitate the understanding of synonymous codon usage patterns and factors contributing its diversification in presumed ancestors of photosynthetic endosymbionts of *P*. *chromatophora*.

## Background

Nucleotide triplet codons, differing only at the third site or rarely at second site but encoding same amino acid are termed as synonymous codons (Ermolaeva 2001). Synonymous mutations do not alter amino acid sequences, but usage of synonymous codons is not at uniform frequencies both within and between organisms, resulting in species specific codon usage bias (Grantham et al. 1980; Sharp et al. 1995). Synonymous codon usage (SCU) bias favours the usage of specific subset of certain codons (preferred codons) within each amino acid family (Agashe et al. 2013). Weak selection of preferred codons has been recognized as an important evolutionary force (Carlini et al. 2001) as SCU bias affects overall fitness of a cell by influencing the level of gene expression and various cellular processes such as RNA processing, translation of protein and protein folding (Parmley and Hurst 2007; Hershberg and Petrov 2008; Plotkin and Kudla 2011). Functional integrity of the genetic code is maintained by synonymous codons (Biro 2008). Population genetic studies reveal that evolution of biased codon usage is mainly either due to genome wide AT/GC biased mutational pressure or due to weak selection acting on specific subset of codons (preferred codons) (Bulmer 1991; Yang and Nielson 2008; Agashe et al. 2013). Other major factors include interaction between codons and anticodons (Kurland 1993), site-specific codon biases (Smith and Smith 1996), efficacy of replication (Deschavanne and Filipski 1995), usage of codon pairs (Irwin et al. 1995) and evolutionary time scale (Karlin et al. 1998).

Forces that influence evolution of SCU bias in various taxa has been extensively analyzed in various organisms (Ikemura 1982; Moriyama and Powell 1997; Nair et al. 2012; Seva et al. 2012; Sharp and Cowe 1991) as SCU bias has high significance in estimating evolutionary rates and phylogenetic reconstruction (Sarmer and Sullivan 1989; Wall and Herback 2003). Previous studies revealed that biased codon usage is stronger in highly expressed genes as selection pressure may be acting on those genes (Ikemura 1985). However, strength of selection appears to be varying among evolutionarily conserved amino acid residues that exhibit stronger bias. In contrast, evolutionarily variable residues often exhibit less or weaker bias (Akashi 1995; Drummond and Wilke 2008). Mutational pressure is another important factor, shaping SCU variations (Plotkin and Kudla 2011; Akashi 2001). Life style of prokaryotic organisms also play important role in SCU variations (Botzman and Margalit 2011). However, role of physiological processes in framing evolution of biased codon usage is yet to be unravelled (Agashe et al. 2013).

Endosymbiotic associations have significant impacts on cellular evolution and diversity (Bodyl et al. 2007). Extensive research on plastid genomes unravelled that a single primary endosymbiotic event in which a cyanobacteria was acquired by a unicellular eukaryote led to the evolution of plastids (Nowack et al. 2008). In endosymbiosis research, *Paulinella chromatophora*, a filose thecamoeba has been regarded as an outstanding model for primary plastid origin as *P*. *chromatophora* is the only known case of independent primary cyanobacterial acquisition (Chan et al. 2011; Marin et al. 2005; Yoon et al. 2006). Sequencing of chromatophore genome revealed the acquisition of photosynthesis by eukaryotes (Nowack et al. 2008). Chromatophores of *P*. *chromatophora* are monophyletic with α - cyanobacteria (Cyanobium clade) (Marin et al. 2007) unlike plastids that were evolved from β - cyanobacterial ancestor (Nowack et al. 2008).

**Figure 1:**
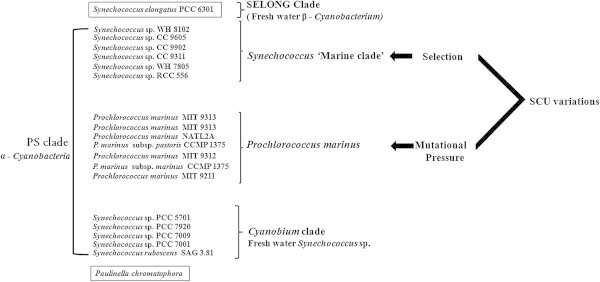
**Diagrammatic representation of three clades in the** ***Prochlorococcus*** **/** ***Synechococcus*** **clade.** SCU variation in marine *Synechococcus* is shaped by selection but in marine *Prochlorococcus*, mutational pressure shapes the SCU pattern. SCU: Synonymous codon usage.

SCU bias in various primary endosymbionts and plastid genomes were extensively studied (Nair et al. 2012; Morton 1993,1997,1998; Sablok et al. 2011). Various factors that frame SCU variations in phylogenetically close marine *Prochlorococcus* and *Synechococcus* clades in the PS clade (*Prochlorococcus*/*Synechococcus*) (Marin et al. 2007) were studied and found that SCU pattern of *Proclorococcus* was shaped by mutational pressure and nucleotide compositional constraints whereas in marine *Synechococcus*, translational selection determine the SCU pattern (Yu et al. 2012). However, no complete cyanobacterial genome has been reported from the Cyanobium clade (third major lineage of PS clade) so far (Figure 1). Hence, comparison of factors that frame SCU in chromatophore genome and its presumed ancestor could not be done. Since habitat of microorganisms play crucial role in SCU variation across genes (Botzman and Margalit 2011), unicellular freshwater β - cyanobacterium *Synechococcus elongatus* PCC6301 (SELONG clade) (Marin et al. 2007) was selected for comparing the SCU patterns and also to elicit the factors determining the SCU variations in evolutionarily young (*P*. *chromarophora*) and evolutionarily old (*S*. *elongatus*) genomes.

## Results

### I. Compositional properties

#### a) Chromatophore genome of *P*. *chromatophora*

Comparison of total A, T, G, C contents in the genome of *P*. *Chromatophora* revealed higher content of A and T than G and C. Analysis of A_3_, T_3_, G_3_, C_3_ contents revealed that T_3_ content was highest and C_3_, the lowest of all with mean and S.D of 39.14% and 4.06% for T_3_ and 12.94% and 3.67% for C_3_. GC_3_ ranged from 16.15% to 54.38% with a mean and S.D of 27.40% and 4.69% respectively. Correlation analysis between total nucleotide contents and silent base contents revealed the stronger negative correlations between A_3_ and GC (Table 1). Similarly, high negative correlation was found between A and GC_3_ (Table 1). This suggests that A and GC contents play important role in SCU bias in the chromatophore genome. High positive correlation between C and G_3_ also might have profound effect in framing SCU patterns. However, no correlations were found between G and T_3,_ and also for T and G_3,_ suggesting no influence of individual T and G contents in codon usage bias. Since A_3_ content was in strong negative correlation with all total nucleotide contents (Table 1), it can be inferred that A_3_ content play an important role in shaping SCU patterns across 786 PCG in the chromatophore genome.

**Table 1:** **Spearman’s rank correlation analysis of nucleotide contents in** ***P. chromatophora***

	A_3_	T_3_	G_3_	C_3_	GC_3_
**A**	0.579**	−0.252**	−0.263**	−0.277**	−0.373**
**T**	−0.158**	0.377**	−0.062	−0.210**	−0.204**
**G**	−0.314**	−0.003	0.358**	0.166**	0.349**
**C**	−0.357**	−0.119**	0.587**	0.117**	0.502**
**GC**	−0.413**	−0.082*	0.323**	0.441**	0.536**

Correlation analysis between total nucleotide contents and silent base contents of 786 PCG in the chromatophore genome of *P. chromatophora.*

*Significant at p ≤ 0.01(one tailed).

**Significant at p ≤ 0.001 (one tailed).

#### b) Genome of *S*. *elongatus*

Contrary to the observations with *P*. *chromatophora*, G and C contents were higher than A and T contents in the genome of *S*. *elongatus*. G_3_ and C_3_ contents were significantly higher than A_3_ and T_3_ contents. Among the silent base contents, C_3_ was highest and A_3_, the lowest of all with mean and S.D of 31.12% and 6.02% for C_3_, and 16.43% and 4.12% for A_3_. GC_3_ varied from 26. 12% to 76.90% with a mean and S.D of 60.19% and 7.45% respectively. Correlation analysis between total A, T, G, C contents and A_3_, T_3_, G_3_, C_3_ contents revealed that A_3_ was negatively correlated to G, C and GC. Similarly, T_3_ was in high negative correlation with G, C, GC_3._ GC composition at silent site was found negatively correlated with both A and T contents (Table 2). Hence, all silent base contents viz., A_3_, T_3_, G_3_ and C_3_ might be influencing SCU variations of protein coding genes (PCG) of *S*. *elongatus*.

**Table 2:** **Spearman’s rank correlation analysis of nucleotide contents in** ***S. elongatus***

	A_3_	T_3_	G_3_	C_3_	GC_3_
**A**	0.522**	0.145**	−0.484**	−0.102**	−0.392**
**T**	0.002	0.618**	−0.151**	−0.364**	−0.382**
**G**	−0.303**	−0.339**	0.671**	0.063*	0.382**
**C**	−0.294**	−0.454**	0.035*	0.559**	0.460**
**GC**	−0.376**	−0.534**	0.482**	0.288**	0.572**

Correlation analysis between total nucleotide contents and silent base contents of 2342 PCG in the *Synechococcus elongatus* PCC 6301.

*Significant at p ≤ 0.01 (one tailed).

**Significant at p ≤ 0.001 (one tailed).

### II. Characteristics of relative synonymous codon usage

#### a) Chromatophore genome of *P*. *chromatophora*

Overall codon usage patterns of 786 PCG in the chromatophore genome of *P*. *chromatophora* were analyzed (Table 3). All the amino acids were found to use A and T ending codons most frequently (codons with RSCU value greater than one) as chromatophore genome is rich in AT than GC. All C ending codons except AGC codes for Ser and CGC codes for Arg and all G ending codons except TTG for Leu were found rare (RSCU values less than 0.66). CTA codes for Leu was the only intermediate codon (RSCU value falls between 0.66 and 1) among the A ending codons. Among the 786 PCG in the chromatophore genome of *P*. *chromatophora*, ENC values ranged from 33.43 to 61 with a mean and S.D of 47.57 and 3.77 respectively, indicating considerable variation in codon usage among the genes of this organism. GC_3_ values ranged from 16.2% to 54.40% with mean and S.D of 27.40% and 4.69% respectively. Chi-square analysis of codon count revealed that 5% of the genes were placed on either side of axis 1, revealing 16 codons were statistically over represented (putative optimal codons) in genes located on the extreme left of the axis 1. Among these codons, ten A ending codons and six T ending codons were found to represent 62.5% A ending codons and 37.5% T ending codons. It is interesting to note that most of the over represented T ending codons were found in 2 codon families except for Glu in which CAA was over represented statistically. These result suggested that some other factors apart from compositional constraints might be influencing the codon usage in this organism.

**Table 3:** **Overall codon usage in** ***P. chromatophora***

AA	Codon	N (RSCU)	AA	Codon	N (RSCU)
Phe	**TTT**	**6873(1.44)**	Tyr	**TAT**	**4818(1.45)**
	TTC	2665(0.56)		TAC	1810(0.54)
Leu	**TTA**	**10679(2.06)**	TER	**TAA**	**439(1.67)**
	TTG	B4586(0.88)		TAG	144(0.55)
	CTT	6933(1.33)	His	**CAT**	**4076(1.54)**
	CTC	2001(0.39)		CAC	1209(0.45)
	CTA	5010(0.96)	Gln	**CAA**	**7465(1.42)**
	CTG	1961(0.38)		CAG	2993(0.57)
Ile	ATC	3060(0.46)	Asn	**AAT**	**8988(1.56)**
	**ATT**	**9871(1.48)**		AAC	2507(0.43)
	ATA	6960(1.05)	Lys	**AAA**	**8697(1.42)**
Met	ATG	5377(1.00)		AAG	3498(0.57)
Val	GTT	5473(1.33)	Asp	**GAT**	**9705(1.60)**
	GTC	2078(0.50)		GAC	2379(0.39)
	**GTA**	**6876(1.68)**	Glu	**GAA**	**11405(1.46)**
	GTG	1941(0.47)		GAG	4189(0.53)
Ser	TCT	4800(1.55)	Cys	**TGT**	**2292(1.42)**
	TCC	1460(0.47)		TGC	922(0.57)
	TCA	3346(1.08)	TER	TGA	203(0.77)
	TCG	912(0.34)	Trp	TGG	3689(1.00)
Pro	**CCT**	**5240(1.80)**	Arg	**CGT**	**5325(2.08)**
	CCC	1350(0.46)		CGC	1765(0.69)
	CCA	4110(1.41)		CGA	2696(1.01)
	CCG	916(0.31)		CGG	892(0.35)
Thr	**ACT**	**5928(1.79)**	Ser	**AGT**	**5768(1.87)**
	ACC	1789(0.54)		AGC	2262(0.73)
	ACA	4202(1.27)	Arg	AGA	3524(1.38)
	ACG	1300(0.39)		AGG	1169(0.46)
Ala	**GCT**	**9187(1.85)**	Gly	**GGT**	**7794(1.64)**
	GCC	2787(0.56)		GGC	2836(0.59)
	GCA	6221(1.25)		GGA	6200(1.30)
	GCG	1670(0.33)		GGG	2129(0.44)

Overall codon usage of 768 PCG in the chromatophore genome of *P. chromatophora.*

Data represented with bold letters are preferred codons.

#### b) Genome of *S*. *elongatus*

Overall codon usage patterns of 2342 PCG in the genome of *S*. *elongatus w*ere analyzed (Table 4). All amino acids except two fold degenerate Phe, Glu, Asp and Lys used G or C ending codons most frequently whereas Phe used TTT, Glu used GAA, Asp used GAT and Lys used AAA most often. Rare codons were TTA, CTT and CTA for Leu, ATA for Ile, GTA for Val, ACA for Thr and GGA for Gly. Intermediate codons were found to be A or T ending predominantly except ACG for Thr, AAG for Lys, GAC for Asp, GAG for Glu, AGG for Arg and GGG for Gly. Among the 14 statistically over represented codons of genes in the extreme left of the axis 1, eight C (56.8 %) ending codons and six G (44.2 %) ending codons were present (Table 5). For 2342 PCG in *S*. *elongatus g*enome, *E*NC values varied from 39.80 to 56.65 with a mean and S.D of 51.29 and 2.14 respectively indicating marked variation in the codon usage of genes in the genome of *S*. *elongatus*. GC_3_ varied from 26.12% to 76.90% with a mean and S.D of 60.19% and 7.45% respectively, suggesting the major influence of GC compositional constraints in framing codon usage across genes in this genome.

**Table 4:** **Overall codon usage in** ***S. elongatus***

AA	Codon	N (RSCU)	AA	Codon	N (RSCU)
Phe	**TTT**	**14909(1.11)**	Tyr	TAT	8278(0.87)
	TTC	11811(0.88)		**TAC**	**10678(1.12)**
Leu	TTA	6634(0.50)	TER	TAA	776(0.96)
	TTG	19535(1.49)		TAG	922(1.15)
	CTT	7121(0.41)	His	CAT	6464(0.96)
	CTC	20585(1.19)		**CAC**	**6949(1.03)**
	CTA	8678(0.50)	Gln	CAA	23338(0.98)
	**CTG**	**32548(1.88)**		**CAG**	**24092(1.01)**
Ile	**ATC**	**21038(1.50)**	Asn	AAT	10381(0.97)
	ATT	20131(1.44)		**AAC**	**10858(1.02)**
	ATA	692 (0.05)	Lys	**AAA**	**10248(1.04)**
Met	ATG	11456(1.00)		AAG	9370(0.95)
Val	GTT	12236(0.94)	Asp	**GAT**	**25896(1.30)**
	GTC	17788(1.37)		GAC	13769(0.69)
	GTA	3535(0.27)	Glu	**GAA**	**24927(1.13)**
	**GTG**	**18179(1.40)**		GAG	18827(0.86)
Ser	TCT	4946(0.81)	Cys	TGT	3451(0.82)
	TCC	5912(0.97)		**TGC**	**4905(1.17)**
	TCA	4257(0.70)	TER	TGA	707(0.882)
	**TCG**	**9207(1.51)**	Trp	TGG	13533(1.00)
Pro	CCT	7920(0.74)	Arg	CGT	7985(0.62)
	**CCC**	**14372(1.35)**		**CGC**	**23748(1.87)**
	CCA	7251(0.68)		CGA	7940(0.62)
	CCG	13017(1.22)		CGG	11080(0.87)
Thr	ACT	7789(0.77)	Ser	AGT	9128(0.82)
	**ACC**	**15479(1.54)**		AGC	13096(1.17)
	ACA	5513(0.55)	Arg	AGA	1135(1.17)
	ACG	11432(0.77)		AGG	791(0.82)
Ala	GCT	19671(0.96)	Gly	GGT	14414(1.02)
	**GCC**	**25932(1.27)**		**GGC**	**24840(1.77)**
	GCA	14455(0.70)		GGA	6797(0.48)
	GCG	21572(1.05)		GGG	9975(0.71)

Overall codon usage of 2342 PCG in the cyanobacterial genome of *S. elongatus.*

Date represented in bold letters are preferred codons.

**Table 5:** **Putative optimal codons**

***Paulinella chromatophora***					***Synechococcus elongatus***						
AA	Codon		AA	Codon		AA	Codon		AA	Codon	
Phe	TTT	**	Tyr	TAT	**	Phe	TTT		Tyr	TAT	
	TTC			TAC			TTC	**		TAC	**
Leu	TTA	**	TER^a^	TAA		Leu	TTA		TER^a^	TAA	
	TTG			TAG			TTG			TAG	
	CTT		His	CAT	**		CTT		His	CAT	
	CTC			CAC			CTC			CAC	**
	CTA		Gln	CAA	**		CTA		Gln	CAA	
	CTG			CAG			CTG	**		CAG	
Ile	ATC		Asn	AAT	**	Ile	ATC	**	Asn	AAT	
	ATT			AAC			ATT			AAC	
	ATA	**	Lys	AAA	**		ATA		Lys	AAA	
Met	ATG			AAG		Met	ATG			AAG	
Val	GTT		Asp	GAT	**	Val	GTT		Asp	GAT	
	GTC			GAC			GTC			GAC	**
	GTA		Glu	GAA	**		GTA		Glu	GAA	
	GTG			GAG			GTG	**		GAG	
Ser	TCT		Cys	TGT	**	Ser	TCT		Cys	TGT	
	TCC			TGC			TCC			TGC	**
	TCA	**	TER	TGA			TCA		TER	TGA	
	TCG		Trp	TGG			TCG	**	Trp	TGG	
Pro	CCT		Arg	CGT		Pro	CCT		Arg	CGT	
	CCC			CGC			CCC			CGC	**
	CCA	**		CGA			CCA			CGA	
	CCG			CGG			CCG	**		CGG	
Thr	ACT		Ser	AGT		Thr	ACT		Ser	AGT	
	ACC			AGC			ACC			AGC	
	ACA	**	Arg	AGA			ACA		Arg	AGA	
	ACG			AGG			ACG	**		AGG	
Ala	GCT		Gly	GGT		Ala	GCT		Gly	GGT	
	GCC			GGC			GCC	**		GGC	**
	GCA	**		GGA	**		GCA			GGA	
	GCG			GGG			GCG			GGG	

Putative optimal codons in *P. chromatophora* and *S. elongatus.*

**Putative optimal codons.

^a^Canonical stop codons excluded from the analysis.

Figures are significant at p ≤ 0.001 (one tailed).

### II. Influence of GC composition on SCUO

#### a) Chromatophore genome of *P*. *chromatophora*

**Figure 2:**
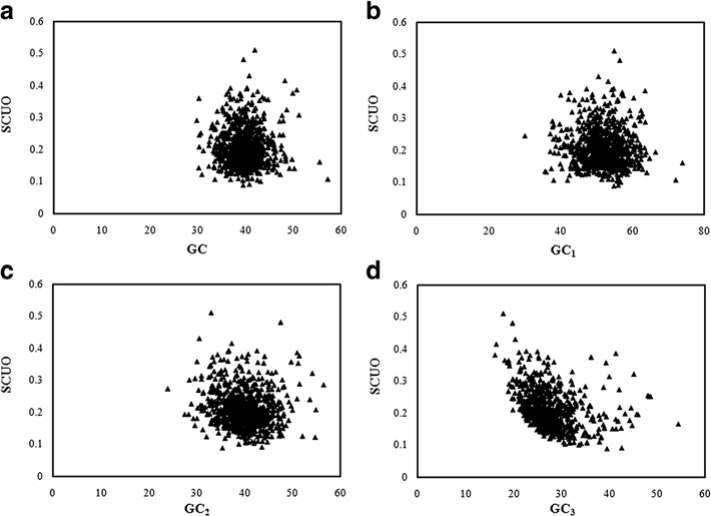
**Relationship between SCUO and GC composition in** ***P. chromatophora*** **. (a)** Relationship between SCUO and the overall GC composition, **(b)** Relationship between SCUO and GC_1_, **(c)** Relationship between SCUO and GC_2_, **(d)** Relationship between SCUO and GC_3_. SCUO: Synonymous codon usage order.

Overall GC content and local GC compositions (GC_1,_ GC_2,_ and GC_3)_ of 786 PCG were estimated and plotted against corresponding SCUO (Figure 2). GC_3_ showed two horns (Figure 2d) whereas overall GC and other local GC compositions (GC_1_ and GC_2)_ did not show any horns. The relationship between GC_3_ and SCUO was found to be linear (SCUO = −0.004 (GC_3)_ + 0.324, r = −0.325, p < 0.001). It was also observed that GC_2_ content was significantly correlated with SCUO values (r = − 0.114, p < 0.001). These results suggested that GC_3_ was more important than GC, GC_1,_ GC_2_ in shaping SCU bias. Thus, mutational bias has important role in SCU variation in chromatophore genome of *P*. *chromatophora*.

#### b) Genome of *S*. *elongatus*

**Figure 3:**
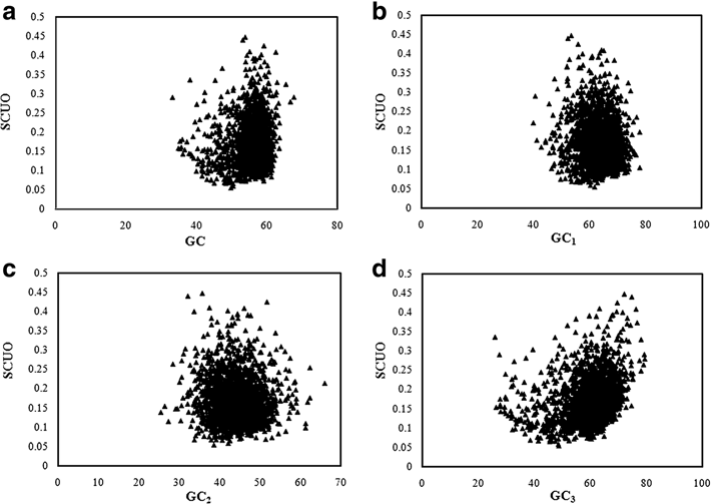
**Relationship between SCUO and GC composition in** ***S. elongatus.*** **(a)** Relationship between SCUO and the overall GC composition, **(b)** Relationship between SCUO and GC_1_, **(c)** Relationship between SCUO and GC_2_, **(d)** Relationship between SCUO and GC_3_.

In the genome of *S*. *elongatus*, total GC content and GC compositions at three codon positions (GC_1,_ GC_2,_ and GC_3)_ were calculated and plotted against corresponding SCUO (Figure 3). GC and GC_3_ showed two horns (Figures 3a and d). SCUO was positively correlated with GC (r =0.063, p < 0.01) and with GC_3_ (r = 0.308, p < 0.001), but negatively correlated with GC_1_ (r = −0.113, p < 0.001) and with GC_2_ (−0.08, p < 0.001), indicating the profound influence of GC_1_ and GC_2_ in SCU variations. In *S*. *elongatus* genome, relationship between SCUO and GC_3_ was found to be linear (SCUO = 0.001(GC_3)_ + 0.052, r = 0.308, p < 0.001). It could be possible that GC_3_ has more influence in SCU variation than other local GC compositions as GC_3_ exhibited the highest correlation with SCUO. Hence, GC mutational pressure may be the key factor that shapes the SCU variation in *S*. *elongatus* genome.

### IV. ENC Vs GC_3_ plot

#### a) Chromatophore genome of *P*. *chromatophora*

**Figure 4:**
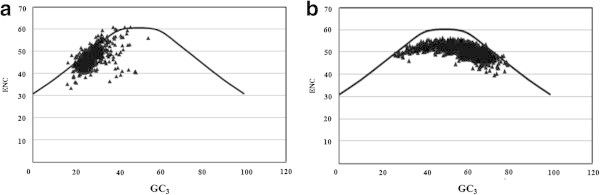
**ENC Vs GC**_**3**_ **plots. (a)** ENC Vs GC_3_ plot of 768 PCG in *P. chromatophora.* **(b)** ENC vs GC_3_ plot of 2342 PCG in *S. elongatus* genome. ENC: Effective number of codons.

**Figure 5:**
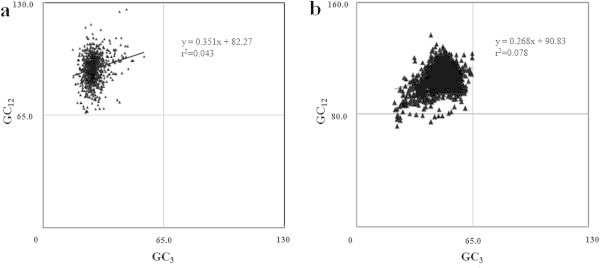
**Neutrality plots. (a)** Neutrality plot of 768 PCG in *P. chromatophora*. **(b)** Neutrality plot of 2342 PCG in *S. elongatus.*

**Figure 6:**
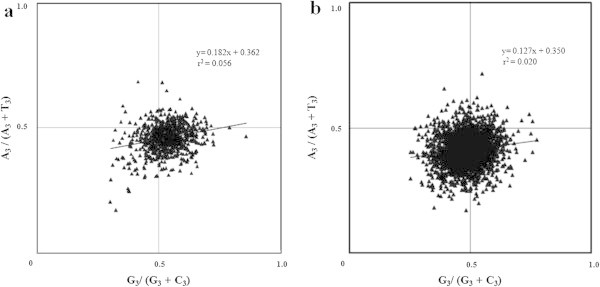
**PR2 bias plots. (a)** PR2 bias plot of 768 PCG in *P. chromatophora.* **(b)** PR2 bias plot of 2342 PCG in *S. elongatus* genome.

ENC Vs GC_3_ plots are generally used for analyzing SCU patterns across genes as axes of this plot are independent of the data and displays intraspecific and interspecific SCU patterns (Wright 1990). If a particular gene is under GC_3_ compositional constraints, it lie on or just below the expected GC_3_ curve. If the SCU pattern of a gene is influenced by translational selection, then it lie considerably below the GC_3_ curve (Wright 1990). ENC values of 786 PCG were plotted against corresponding GC_3_ values (Figure 4a) and majority of the genes were clustered on the left side of the curve. Though some genes lie on or just below the expected GC_3_ curve, most of the genes were clustered below the curve. This indicated the influence of certain forces other than GC_3_ compositional constraints in shaping SCU patterns in chromatophore genome of *P*. *chromatophora*. Significant correlation observed between GC_12_ and GC_3_ (r = 0.207, p < 0.001) in neutrality plot (Figure 5a) has nullified the influence of selection in framing the codon usage pattern of chromatophore genes. Further, influence of GC_3_ mutational pressure on PCG was analyzed using PR2 bias plot (Figure 6a) and observed that synonymous A, T and G, C contents were used proportionally (y = 0.182x + 0.362, r = 0.236), confirming the role of GC_3_ biased mutational pressure in shaping the SCU across 786 PCG in the chromatophore genome of *P*. *chromatophora*.

#### b) Genome of *S*. *elongatus*

Majority of the genes were grouped considerably below the expected GC_3_ curve (Figure 4b), indicating the influence of some other forces other than GC compositional constraints. In neutrality plot (Figure 5b), GC_12_ was significantly correlated with GC_3,_ indicating that selection has only weak role in SCU variation. The influence of GC_3_ on SCU variation was analyzed by PR2 bias plot (Figure 6b) and revealed that A, T and G, C contents were used proportionally (y = 0.127 + 0.350, r = 0.140), reflecting the GC_3_ compositional constraints in SCU variation across 2342 PCG in the *S*. *elongatus* genome.

### V. Correspondence analysis (COA)

#### a) Chromatophore genome of *P*. *chromatophora*

**Figure 7:**
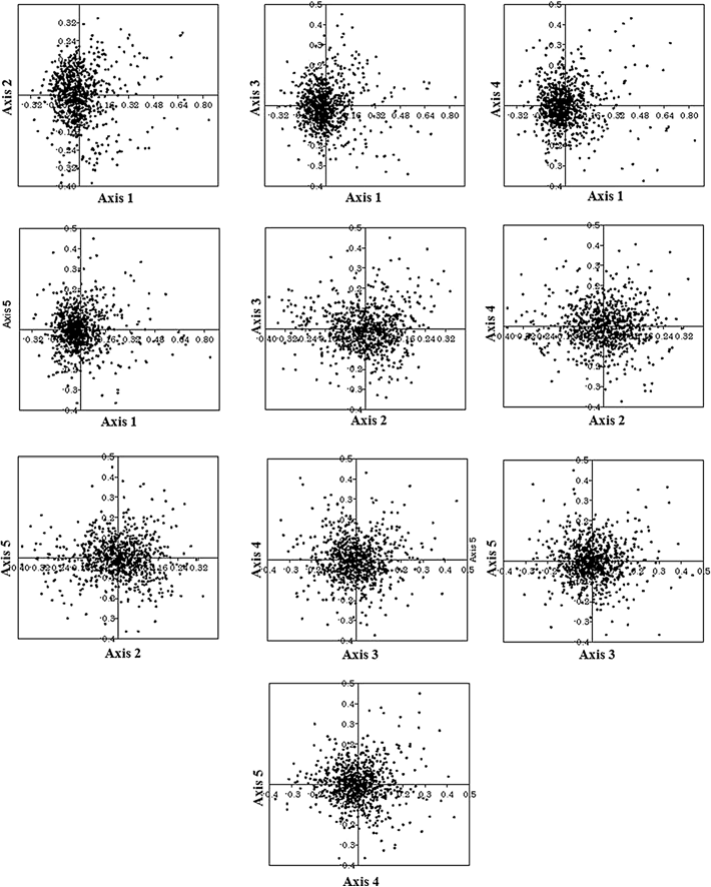
**Correspondence analysis.** Correspondence analysis on RSCU values of 768 PCG in the chromatophore genome of *P. chromatophora.*

Axis 1, axis 2, axis 3, axis 4 and axis 5 accounted for 7.31%, 5.15%, 4.43%, 4.32% and 3.89% of total variations respectively (Figure 7). No single major explanatory axis was identified for explaining the variations. Spearman’s rank correlation analysis between five axes of COA and various indices of codon usage revealed that all axes except axis 3 and 5 were in significant correlation with silent base contents (Table 6). For instance, axis 1 with A_3,_ G_3,_ C_3,_ axis 2 with A_3,_ T_3,_ and axis 4 with A_3,_ T_3,_ C_3,_ GC_3._ Strong negative correlation existed between axes 1 and 2 with A_3,_ and axis 4 with T_3_ suggested the influence of compositional constraints in shaping codon usage of chromatophore genes. Complex correlations were observed among 59 synonymous codons and five axes of COA. Interestingly, Cys codons (TGT and TGC) were found to have the highest correlation with axis 2 (Table 7). Thus, Cys codons may have high influence in separating PCG along axis 2. Axes 1 and 4 shown significant negative correlation with ENC and CAI. Hence, it could be assumed that genes, distributed along axes 1 and 4 might be influenced by some amount of selection. Length of CDS was found to be in correlation only with axis 1. Since axis 1 did not account for much of the variations, length of CDS could not be considered as an important factor that frames SCU across genes. Aromaticity and protein gravy scores were not correlated with any one of the axes, indicating no influence in shaping codon usage patterns of chromatophore genes in the *P*. *chromatophora*.

**Table 6:** **Spearmen’s rank correlation analysis between COA axes and codon usage indices**

Axes	A_3_	T_3_	G_3_	C_3_	GC_3_	ENC	CAI	Gravy score	Aromaticity	Length of CDS
**Axis 1**	−0.434**	−0.094	0.221**	0.559**	0.565**	0.345**	−0.360**	−0.064	−0.081	−0.140**
**Axis 2**	−0.159**	0.118**	0.092	−0.014	0.041	−0.072	0.104	−0.045	−0.028	0.006
**Axis 3**	0.008	0.016	0.057	−0.057	−0.015	0.044	0.063	−0.023	−0.024	0.065
**Axis 4**	0.173**	−0.404**	0.125	0.167**	0.187**	0.343**	−0.258**	0.043	−0.028	−0.074
**Axis 5**	−0.027	0.060	−0.031	−0.006	−0.035	−0.031	−0.022	−0.075	−0.030	−0.096

Correlation analysis between five different axes of COA and various codon usage indices of 786 PCG in the chromatophore genome of *P. chromatophora.*

Analysis was made using Spearman’s rank correlation method.

**Significant at p ≤ 0.001 (one tailed).

**Table 7:** **Correlation analysis between COA axes and synonymous codons**

***P. chromatophora***							***S. elongates***			
Codons	Axis 1	Axis 2	Axis 3	Axis 4	Axis 5	Axis 1	Axis 2	Axis 3	Axis 4	Axis 5
GCT	0.017	0.093*	−0.021	0.077	0.025	0.213**	−0.094*	0.013	0.249**	−0.064
GCG	0.027	0.019	−0.041	−0.046	0.057	−0.161**	0.111**	0.019	−0.334**	0.136**
GCC	0.156**	0.017	−0.121**	−0.029	0.007	−0.290**	−0.040	−0.072	−0.019	−0.031
GCA	−0.133**	−0.106**	0.130**	0.110**	−0.057	0.279**	0.022	0.054	0.085	−0.067
TGT	−0.416**	0.723**	−0.116**	0.051	−0.090*	0.292**	0.190**	0.188**	−0.477**	−0.619**
TGC	0.291**	−0.792**	0.192**	−0.049	−0.003	−0.239**	−0.089**	−0.247**	0.371**	0.686**
GAT	−0.286**	−0.064	0.035	−0.099**	0.058	0.257**	0.189**	0.158**	−0.215**	0.128**
GAC	0.290**	0.058	−0.032	0.093**	−0.051	−0.256**	−0.188**	−0.166**	0.218**	−0.129**
GAG	0.231**	0.015	0.097**	−0.079	−0.061	0.044	0.113**	−0.036	−0.110**	0.037
GAA	−0.238**	−0.022	−0.091*	0.072	0.054	−0.037	−0.116**	0.040	0.106**	−0.028
TTT	−0.266**	−0.087*	0.088*	−0.070	0.083	0.339**	0.195**	0.209**	−0.178**	0.099**
TTC	0.248**	0.082	−0.086*	0.060	−0.083	−0.348**	−0.194**	−0.211**	0.171**	−0.099**
GGT	0.129**	0.083	0.079	−0.030**	−0.028	0.012	−0.128**	−0.005	0.177**	−0.098**
GGG	0.034	−0.122**	0.011	0.096**	−0.021	0.161**	0.201**	0.041	−0.271**	0.110**
GGC	0.220**	0.014	−0.088*	0.050	0.021	−0.403**	−0.137**	−0.097*	0.080	−0.047
GGA	−0.342**	−0.031	−0.023	0.159**	−0.004	0.396**	0.196**	0.109**	−0.102**	0.098**
CAC	0.213**	0.178**	0.600**	0.170**	0.260**	−0.368**	−0.159**	−0.224**	0.051	−0.196**
CAT	−0.278**	−0.213**	−0.530**	−0.152**	−0.299**	0.360**	0.178**	0.187**	−0.107**	0.246**
ATT	−0.050	−0.008	0.015	−0.141**	0.121**	0.267**	0.019	0.138**	−0.057	0.076
ATA	−0.217**	0.037	−0.045	0.251**	−0.095**	0.323**	0.022	−0.039	0.071	0.030
ATC	0.299**	−0.034	0.020	−0.118**	−0.015	−0.341**	−0.022	−0.134**	0.029	−0.085
AAA	−0.219**	−0.207**	0.025	0.068	0.075	0.038	−0.040	0.050	0.176**	−0.061
AAG	0.217**	0.188**	−0.022	−0.077	−0.079	−0.068	0.001	−0.072	−0.167**	0.050
CTA	−0.091*	−0.171**	0.029	0.189**	−0.127**	0.345**	0.102**	0.038	0.026	0.034
CTC	0.125**	0.011	−0.074	0.087*	0.052	−0.079	−0.108**	−0.022	0.088	−0.022
CTG	0.185**	0.008	0.064	0.076	−0.051	−0.403**	0.003	−0.032	−0.218**	0.038
CTT	−0.151**	0.119**	0.004	−0.268**	0.059	0.374**	0.061	0.032	0.153**	−0.060
TTA	−0.190**	−0.041	0.012	−0.036	−0.055	0.481**	0.156**	0.056	−0.008	0.073
TTG	0.144**	0.029	0.006	0.053	0.055	−0.468**	−0.142**	−0.048	0.001	−0.071
AAC	0.291**	0.036	0.117**	0.055	−0.045	−0.383**	−0.257**	−0.189**	0.188**	−0.167**
AAT	−0.291**	−0.029	−0.115**	−0.068	0.045	0.382**	0.240**	0.163**	−0.180**	0.165**
CCA	−0.248**	−0.171**	0.215**	0.113**	−0.369**	0.387**	0.159**	0.080	−0.029	0.094*
CCC	0.252**	0.031	0.009	0.306**	0.379**	−0.247**	−0.114**	−0.113**	0.017	−0.006
CCT	0.053	0.142**	−0.315**	−0.321**	0.075	0.304**	0.038	0.085	0.206**	−0.110**
CCG	0.003	−0.031	0.259**	−0.007	−0.011	−0.341**	−0.009	−0.057	−0.231**	0.051
CAA	−0.255**	−0.123**	−0.041	0.075	0.075	0.085	−0.116**	0.038	0.210**	−0.040
CAG	0.255**	0.123**	−0.041	−0.075	−0.075	−0.085	0.116**	−0.038	−0.210**	0.040
AGA	−0.049	−0.261**	−0.216**	−0.332**	0.502**	0.552**	−0.452**	−0.606**	−0.173**	−0.022
AGG	−0.100**	0.221**	0.244**	0.349**	−0.489**	0.261**	0.670**	−0.471**	0.037	−0.062
CGA	−0.357**	−0.197**	−0.140**	0.433**	0.246**	0.448**	0.174**	0.042	−0.176**	0.152**
CGC	0.373**	0.066	−0.413**	0.178**	−0.185**	−0.330**	−0.031	−0.018	−0.009	0.030
CGG	−0.046	0.048	0.138**	0.091	0.039	−0.067	0.029	−0.019	−0.193**	−0.002
CGT	0.048	0.107**	0.395**	−0.599**	−0.107**	0.062	−0.114**	−0.027	0.305**	−0.156**
AGC	0.248**	−0.005	−0.027	0.064	−0.174**	−0.331**	−0.237**	−0.161**	0.200**	−0.129**
AGT	−0.263**	−0.002	0.028	−0.053	0.182**	0.338**	0.242**	0.160**	−0.206**	0.126**
TCA	−0.275	−0.248	0.127	0.132	−0.178	0.365**	0.086	0.092	0.050	0.267**
TCC	0.145**	0.020	−0.143**	0.212**	−0.096**	−0.225**	−0.109**	−0.170**	0.016	−0.203**
TCG	−0.048	0.114**	−0.141**	0.020	0.048	−0.353**	0.067	−0.045	−0.450**	0.187**
TCT	0.128**	0.169**	0.068	−0.311**	0.191**	0.341**	−0.015	0.081	0.411**	−0.261**
ACC	0.299**	−0.053	−0.090*	−0.590**	−0.276**	−0.438**	−0.185**	−0.088	0.166**	−0.077
ACA	−0.325**	−0.057	0.185**	0.083	0.002	0.403**	0.178**	0.020	−0.010	−0.004
ACG	−0.057	−0.080	−0.048	0.119**	0.254**	−0.189**	0.061	0.011	−0.297**	0.151**
ACT	0.137**	0.148**	−0.090*	−0.139**	0.018	0.366**	0.014	0.054	0.087	−0.057
GTT	0.039	−0.073	0.071	−0.176**	0.003	0.361**	−0.020	0.061	0.190**	−0.029
GTG	0.164**	0.047	−0.019	0.154**	−0.040	−0.239**	0.098	0.025	−0.268**	0.055
GTC	0.027	0.074	−0.130**	0.145**	0.143**	−0.241**	−0.119**	−0.102**	0.112**	−0.026
GTA	−0.166**	−0.050	0.045	−0.044	−0.100**	0.226**	0.070	0.005	−0.072	−0.025
TAC	0.273**	0.002	−0.015	0.162**	−0.246**	−0.443**	−0.215**	−0.178**	0.223**	−0.113**
TAT	−0.277	−0.017	−0.012	−0.184	0.223	0.423**	0.217**	0.159**	−0.242**	0.114**

Correlation analysis between five different axes of COA and 59 synonymous codons in chromatophore genome and *S. elongatus* genome.

Analysis was made using Spearman’s rank correlation method.

*Figures are significant at p ≤ 0.01 (one tailed). **Figures are significant at p ≤ 0.001 (one tailed).

#### b) Genome of *S*. *elongatus*

**Figure 8:**
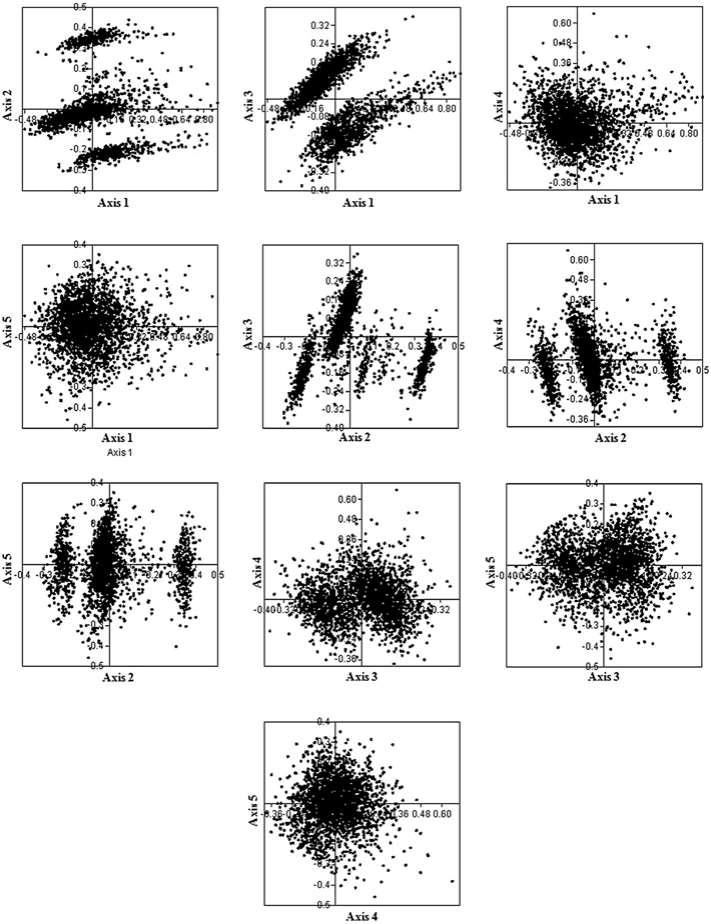
**Correspondence analysis.** Correspondence analysis on RSCU values of 2342 PCG in *S. elongatus.*

Axis 1, axis 2, axis 3, axis 4 and axis 5 accounted for 12.22%, 7.93%, 5.24%, 4.80% and 4.30% of total variations respectively (Figure 8). None of the axes was found to contribute majority of variation. All PCG were found to be separated into three clusters along axis 2. All C ending codons were found to have strong negative correlation with axis 2. Clusters were formed based on the RSCU value of each C ending codons. Correlation analysis was performed between various axes of COA and codon usage indices (Table 8). However, axes 1, 2, 3, and 4 were in significant negative correlation with GC_3._ Interestingly, axes 1, 2 and 3 were negatively correlated with length of CDS. Thus GC_3_ compositional constraints and length of CDS might be influencing the SCU patterns across genes in the *S*. *elongatus* genome. Among the silent base contents and various axes of COA, positive correlation existed between axis 1 with A_3_ and T_3,_ axis 2 with A_3,_ T_3,_ and G_3,_ axis 3 with A_3_ and T_3,_ axis 4 with A_3,_ T_3_ and C_3_ and axis 5 with A_3._ This suggested the influence of nucleotide compositional constraints in SCU variation in *S*. *elongatus* genome. ENC was positively correlated with axes 1, 2, and 3 whereas CAI was in positive correlation with axis 1, but negatively correlated with axis 3. Thus, weak selection might influence the SCU of genes in *S*. *elongatus*. Axes 2 and 3 were positively correlated with protein gravy score, but axis 4 was negatively correlated, indicating the possible influence of hydropathic character of protein in SCU variation across genes in *S*. *elongatus* genome.

**Table 8:** **Correlation analysis between COA axes and codon usage indices**

Axes	A_3_	T_3_	G_3_	C_3_	GC_3_	ENC	CAI	Gravy score	Aromaticity	Length of CDS
**Axis 1**	0.616**	0.591**	−0.224**	−0.674**	−0.761**	0.588**	0.076**	−0.016	0.018	−0.050*
**Axis 2**	0.095**	0.086**	0.221**	−0.296**	−0.119**	0.138**	0.068**	0.063*	0.001	0.029
**Axis 3**	0.107**	0.212**	0.033	−0.025	−0.212**	0.076**	−0.086**	0.096**	0.003	−0.198**
**Axis 4**	0.053*	0.113**	−0.508**	0.267**	−0.090**	−0.023	0.016	−0.096**	0.065*	−0.137**
**Axis 5**	0.064*	−0.059*	0.168**	−0.143**	0.003	0.002	−0.036	0.017	0.040	0.044*

Correlation analysis between five different axes of COA and various codon usage indices of 2342 PCG in the cyanobacterial genome of *S. elongatus.*

Analysis was made using Spearman’s rank correlation method.

*Figures are significant at p ≤ 0.01 (one tailed).

**Figures are significant at p ≤ 0.001 (one tailed).

## Discussion

Chromatophore genome of *P*. *chromatophora* has typical cyanobacterial characteristics (Yoon et al. 2006) as *P*. *chromatophora* was diverged as sister to free living α - cyanobacteria (Marin et al. 2007). It was proposed that photosynthetic endosymbionts of *P*. *chromatophora* were evolved from cyanobium clade (Marin et al. 2007) which is paradoxical to the previous finding that chromatophores were evolved from the marine clade, consisting *Prochlorococcus* and *Synechococcus* (Marin et al. 2005). However, no complete cyanobacterial genome was reported so far from freshwater α-cyanobacteria in the cyanobium clade to compare various factors that shape SCU variation in photosynthetic endosymbionts (chromatophores) of *P*. *chromatophora* and its presumed ancestor genome. In this context, SCU patterns and factors contributing diversification in the genomes of chromatophore and freshwater unicellular β – cyanobacterium *S*. *elongatus* (SELONG clade) (Marin et al. 2007) were studied. The present findings revealed that mutational pressure due to GC compositional constraints frame the SCU patterns in both genomes but with varying intensity. Factors influencing SCU variation in marine *Prochlorococcus* and S*ynechococcus* (Yu et al. 2012) from the PS clade (Marin et al. 2007) revealed that mutational pressure plays important role in SCU variation of *Prochlorococcus* but for *Synechococcus*, selection dictates the SCU pattern. In the present study, ENC Vs GC_3_ plots of chromatophore genes and genes of freshwater *S*. *elongatus* showed that majority of genes were clustered on or just below the expected curve as observed in the ENC Vs GC_3_ plot of genes of *Prochlorococcus* genome (Yu et al. 2012). Whereas, only few genes of marine *Synechococcus* genome were lying on or just below the expected curve indicating the influence of some additional factors in framing codon usage patterns (Yu et al. 2012). Variation of factors influencing SCU patterns in fresh water *Synechococcus* sp. and marine *Synechococcus* sp. reveals that life pattern of organisms may diversify the factors contributing SCU variation even within the same genus, supported by the previous observation that evolution of microbe is very often influenced either by environment or by life style (Botzman and Margalit 2011;Paul et al. 2010).

Putative optimal codons, detected in chromatophore and *S*. *elongatus* genome are of great importance as they improve expression of heterologous genes in host cells (Wang et al. 2013). Equilibrium between neutral mutational pressure and natural selection is important in maintaining the heterogeneity of codon usage among species (Sueoka 1988) and if significant correlation exists between GC_12_ and GC_3,_ it can be assumed that codon usage pattern is mainly framed by mutational pressure and if no such correlation exists, translational selection would be the major force. In the present study, neutrality plot revealed significant correlations between GC_12_ and GC_3_ of genes from chromatophore and genome of *S*. *elongatus*. Most of the 786 PCG of chromatophore and 2342 PCG of S. *elongatus* were grouped on the upper left of the neutrality plot. Slope of the regression line in both plots were not close to zero, indicating that influence of specific evolutionary pressure such as selection is weak. Thus, it can be proposed that mutational pressure is the key factor that shapes the codon usage pattern of both chromatophore and *S*. *elongatus* genome. Moreover, in PR2 bias plot of these two genomes, synonymous A, T and G, C contents were found to be used proportionally indicating the influence of GC compositional constraints. Interestingly, in the PS clade, significant correlation between GC_12_ and GC_3_ was found only in *Prochlorococcus* (Yu et al. 2012). Thus, we can assume that freshwater *P*. *chromatophora* genome and *S*. *elongatus* genome are more similar to Marine *Prochlorococcus* than Marine *Synechococcus* in terms of factors that diversify SCU patterns.

Relationship between SCUO and GC_3_ formed a ‘U’ shape with two horns in both genomes as reported in unicellular microorganisms (Wan et al. 2004) and it reveals the influence of GC_3_ over SCU bias. In chromatophore genome, three axes of COA were found to show higher correlation with silent base contents, confirming the influence of genome wide compositional constraints. However, axes 1 and 4 were highly correlated with codon usage indices that indicate the level of gene expression such as ENC and CAI. Since there were no major explanatory axes, correlation with these indices cannot be linked with the influence of selection. Hydropathic character of protein (gravy score) was correlated with axes 2, 3 and 4 in *S*. *elongatus* genome, suggesting that silent sites may be affected by hydropathy levels of protein whereas in chromatophore genome, gravy score did not show any correlation with any of the axes of COA. Correlation between length of CDS and axes 1, 3 and 4 in *S*. *elongatus* genome indicate the influence of length of CDS in SCU variation but no such correlation was existed in *P*. *chromatophora*. In *S*. *elongatus* genome, negative correlation existed between GC_3_ and first four axes of COA confirms the GC_3_ consequence on SCU pattern. Indices indicating the level of gene expression such as ENC and CAI were correlated significantly with first three axes of COA reflect the weak selection may take part in SCU variation of *S*. *elongatus*. Formation of three clusters of PCG along axis 2 in *S*. *elongatus* genome indicating a trend associated with RSCU value of C ending codons, but not observed in chromatophore genome. Whereas in chromatophore genome, TGT and TGC codons (encoding Cys) influence separation of PCG along axis 2. Influence of Cys codons in shaping SCU pattern was already reported in *Lactococcus lactis* (Gupta et al. 2004) and *Rhizobium* (Wang et al. 2013). However, these results suggested that genome wide compositional constraints influence the SCU patterns of both chromatophore genome and *S*. *elongatus* genome.

SCU patterns of chromatophore genome of *P*. *chromatophora* and *S*. *elongatus* may be closely associated with living habitats. The adapted habitat of *P*. *chromatophora* is a submerged vegetation in freshwater. Mud loving nature of this organism protects it from potential extrinsic mutagens like UV-B radiation and which in turn causes genome wide mutation as reported in *Prochlorococcus* (Partensky et al. 1999). Freshwater β – cyanobacterium *S*. *elongatus* PCC6301 is less adaptive to varying environments as it resides strictly in euphotic zones, relatively with low nutrient contents at mesophilic temperature (Waterbury et al. 1986) unlike marine *Synechococcus* which is more adaptive to grow in varying nutrient conditions and temperatures (Moore et al. 1998). To make marine *Synechococcus* more adaptive to environment, translational selection shapes the codon usage patterns (Yu et al. 2012) but mutational pressure frames codon usage in less adaptive fresh water *S*. *elongatus*. Closely related species, living in distinct environments may exhibit considerable genomic diversity (Paul et al. 2010) that lead to differences in factors behind diversification of SCU patterns. Mutational pressure was found to be the major factor, influencing SCU pattern across PCG in strictly thermophilic cyanobacterium *Thermosynechococcus elongatus* BP-1 (Prabha et al. 2012) which is less adaptive to other temperature ranges as growth of thermophiles is restricted to particular environment at specific temperature (Botzman and Margalit 2011). These reports support our finding that SCU pattern of *P*. *chromatophora* and *S*. *elongatus* is dictated by mutational pressure due to their less adaptation to varying environments.

## Conclusions

SCU pattern of photosynthetic endosymbiont (chromatophore) and *S*. *elongatus* genome is dictated mainly by genome wide GC mutational pressure. Living habitats of *P*. *chromatophora* and *S*. *elongatus* may also be influencing the SCU variations across genes of both genomes. However, complete genome sequencing of α-cyanobacteria from cyanobium clade would help further to understand SCU pattern and factors contributing diversification of SCU in presumed ancestors of photosynthetic endosymbionts of *P*. *chromatophora*.

## Methods

### Gene sequences

Complete coding sequences (CDS) of chromatophore genome (Genbank: NC_011087.1) of *P*. *chromatophora* (Nowack et al. 2008) and genome (Genbank: AP008231) of *S*. *elongatus* (Sugita et al. 2007) were retrieved from NCBI and CYORF (Cyanobacterial gene annotation database) respectively. CDS integrity was confirmed by checking the presence of START codon at the beginning and STOP codon at the end of each codon without any internal stop codons. To minimize the sampling errors, CDS with more than 300 nucleotides were chosen for analysis (Zhou and Li 2009;Sablok et al. 2011). Duplicate sequences were identified and excluded from the data set. Thus, the final data set of chromatophore genome consists 786 coding sequences that contain 2, 61,350 codons and 7, 84,050 nucleotides, whereas final data set of genome of *S*. *elongates* contains 2342 coding sequences that contain 7, 74, 810 codons and 23, 24, 430 nucleotides.

### Indices of codon usage

#### a) Relative synonymous codon usage (RSCU)

To infer the features of SCU variations across PCG in the chromatophore genome by not taking amino acid compositional constraints into account, the RSCU values of all PCG were estimated according to Sharp et al. (1986).

#### b) Effective number of codons (ENC)

ENC is an index that is widely used for measuring the extent of synonymous codon usage bias (Wright 1990). It can take values from 20 (only one codon is used for each of the 20 aminoacids) to 61 (when all synonymous codons are equally used). If the calculated ENC value is beyond 61 due to more even distribution of codon usage, it is adjusted to 61 (Wright 1990). Selection of preferred codons and mutational pressures may reduce the ENC values. The expected ENC under random codon usage is approximated as a function of GC_3_ and calculated according to Wright (1990).

#### c) Codon adaptation index (CAI)

Codon adaptation index (CAI) is a measure of bias towards preferred codons in a PCG by defining the translationally optimal codons that are mostly represented in a reference set of highly expressed genes (Sharp and Li 1987). CAI value ranges from zero to one. Higher value indicates increased bias towards preferred codons. For this study, we used ribosomal protein coding genes as reference for estimating CAI values on the basis of equation, developed by Sharp and Li (1987).

#### d) Synonymous codon usage order (SCUO)

Synonymous codon usage order measurement was used to analyze the influence of GC composition at various codon positions on SCU. SCUO was computed using the following equation (Wan et al. 2004),

### Sequence analysis

Nucleotide contents of all PCG were calculated using MEGA version 5.1 (Tamura et al. 2011). ENC values and CAI were calculated for all PCG by using online CodonW (http://codonw.sourceforge.net) and CAI calculator 2 (Wu et al. 2005). SCUO was computed using standalone CodonO (Wan et al. 2004).

### Correspondence analysis (COA)

COA is a multivariate statistical method used to identify major factors, shaping SCU patterns across genes and plot genes according to various influencing factors of SCU (Perriere and Thioulouse 2002). Multivariate statistical analysis method was often employed to plot PCGs according to RSCU values of the 59 synonymous codons (excluding 3 stop codons, Trp and Met codons) (RoyChoudhury and Mukherjee 2010). COA develops a series of orthogonal axes to define the major factors that frame the SCU patterns in accordance with the variation of data. In this study, complete coding regions of each PCG were represented as a 59 dimensional vector (excluding Met, Trp and stop codons). Each dimension corresponds to RSCU value of one sense codon (Mardia et al. 1979).

### Statistical analysis

All correlations were made using Spearman’s rank correlation method as this measure of correlation does not require any distributional assumptions of the underlying data (Zhou and Li 2009). A Chi - square test involving 2 × 2 table was employed for 5% of genes distributed at extreme left and 5% of genes distributed at extreme right of axis 1 of COA to find out putative optimal codons. For each of 59 sense codons, First row contains the observed frequency of a codon and the second row contains total number of synonymous alternatives of that particular codon. The significance was calculated at the 5% level with one degree of freedom. All these analyses were done using Past version 2.12 (Hammer et al. 2001).

## Abbreviations

PCGs: Protein coding genes
RSCU: Relative synonymous codon usage
SCU: Synonymous codon usage
ENC: Effective number of codon usage
CAI: Codon adaptation index
SCUO: Synonymous codon usage order
PR2: Parity rule 2
GC3: GC content at 3rd codon position
GC12: GC content at first and second position
COA: Correspondence analysis.
